# PKSmart: an open-source computational model to predict intravenous pharmacokinetics of small molecules

**DOI:** 10.1186/s13321-025-01066-5

**Published:** 2025-09-26

**Authors:** Srijit Seal, Maria-Anna Trapotsi, Manas Mahale, Vigneshwari Subramanian, Nigel Greene, Ola Spjuth, Andreas Bender

**Affiliations:** 1 Imaging Platform, Broad Institute of MIT and Harvard, Cambridge, MA USA; 2 Yusuf Hamied Department of Chemistry, University of Cambridge, Lensfield Road, Cambridge, CB2 1EW UK; 3 Imaging & Data Analytics, Clinical Pharmacology & Safety Sciences, AstraZeneca R&D, 1 Francis Crick Way, Cambridge, CB2 0AA UK; 4 Bombay College of Pharmacy Kalina Santacruz (E), Mumbai, 400 098 India; 5 Imaging & Data Analytics, Clinical Pharmacology & Safety Sciences, AstraZeneca R&D, 35 Gatehouse Drive, Waltham, MA 02451 USA; 6 Department of Pharmaceutical Biosciences and Science for Life Laboratory, Uppsala University, Box 591, 75124 Uppsala, Sweden; 7 College of Medicine and Health Sciences, Khalifa University of Science and Technology, 127788 Abu Dhabi, United Arab Emirates; 8 Safety Innovation, Clinical Pharmacology and Safety Sciences, AstraZeneca R&D, Pepparedsleden 1, 43183 Mölndal, Sweden

**Keywords:** Machine learning, Toxicity, Bioactivity, Applicability domain, Pharmacokinetic parameters, Clearance, Volume of distribution

## Abstract

**Abstract:**

Drug exposure, a key determinant of drug safety and efficacy, is governed by pharmacokinetic (PK) parameters such as volume of distribution (VDss), clearance (CL), half-life (t½), fraction unbound in plasma (fu), and mean residence time (MRT). In this study, we developed machine learning models to predict human PK parameters for 1,283 unique compounds using molecular structure, physicochemical properties, and predicted animal PK data. Our approach involved a two-stage modeling pipeline. First, we trained models to predict rat, dog, and monkey PK parameters (VDss, CL, fu) from chemical structure and properties for 371 compounds. These models were used to predict animal PK values for 1,283 unique compounds with human PK data. These animal PK predictions were then integrated with molecular descriptors and fingerprints to build Random Forest models for human PK parameters. The models demonstrated consistent performance across nested cross-validation and external validation sets, with predictive accuracy for VDss comparable to proprietary models developed by AstraZeneca. Notably, human VDss and CL predictions achieved external R^2^ values of 0.39 and 0.46, respectively. To support broad accessibility and integration into early drug discovery workflows such as Design-Make-Test-Analyze (DMTA), we developed PKSmart (https://broad.io/PKSmart), a freely available web application. All code and models are also open source, enabling local deployment. To our knowledge, this represents the first public suite of PK prediction models with performance on par with industry standard models.

**Scientific contribution:**

This study introduces the first publicly available pharmacokinetic (PK) models that match industry-standard predictions, utilizing molecular structural fingerprints, physicochemical properties, and predicted animal PK data to model human pharmacokinetics. Our approach is validated through repeated nested cross-validation and an external test set, including comparing predictions to an industry standard model. The models are released via a web-hosted application (https://broad.io/PKSmart) for wider accessibility and utility in drug development processes.

**Graphical Abstract:**

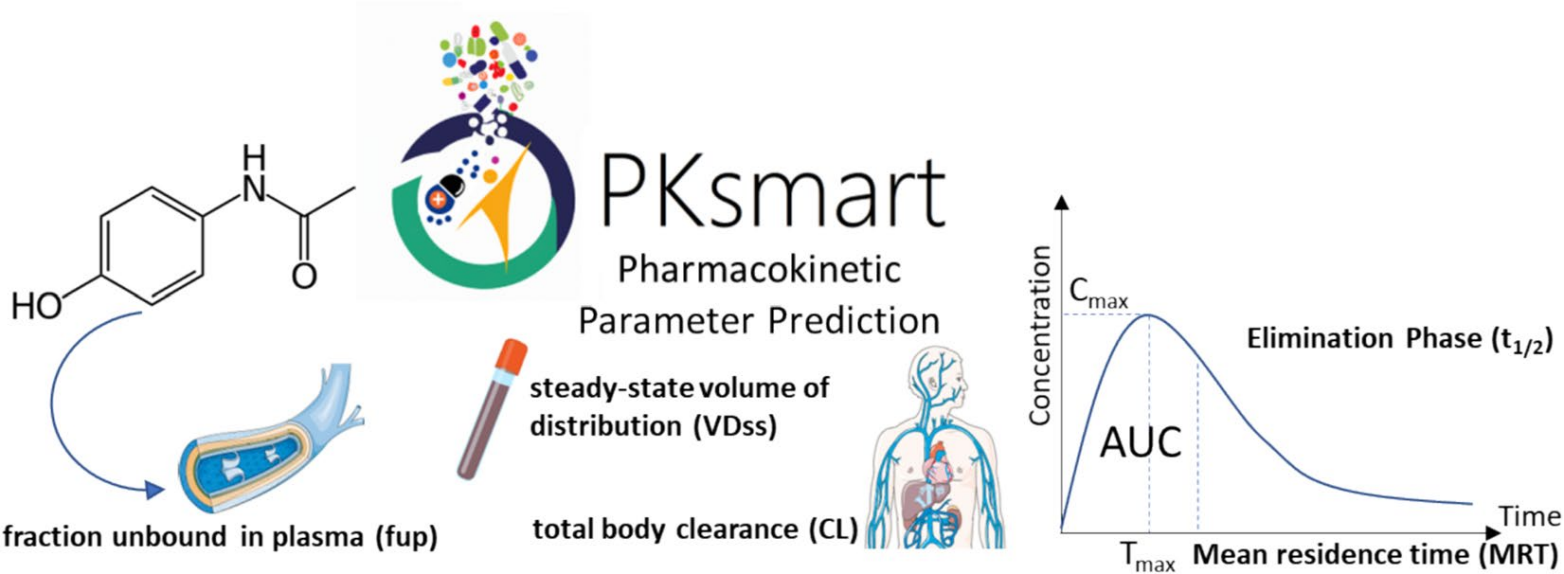

**Supplementary Information:**

The online version contains supplementary material available at 10.1186/s13321-025-01066-5.

## Introduction

The mechanism of action of a compound, especially at an organism level, is not only dependent on the biological activity of the compound but also its exposure [1], which can be defined by factors such as drug bioavailability, the volume of distribution, and clearance among other parameters [2]. One way to estimate drug exposure *in vivo* is the extrapolation from *in vitro* data (also known as IVIVE: *in vitro* to *in vivo* extrapolation). However, predictions for *in vivo* intrinsic clearance tend to be underestimated for drugs with high observed *in vivo* intrinsic clearance [3]. Other methods of estimating pharmacokinetics (PK) parameters include allometric scaling [4] (or single-species scaling) and physiologically based pharmacokinetic modelling (PBPK) [5]. Previously, simple allometry and interspecies scaling [6] have been used for the prediction of PK parameters, such as CL with an average fold error < 2.0 for small molecules [7]. Allometric relations between rat and human CL, VDss and t½ require measured PK parameters and have been reasonably accurate [4]; however, they sometimes have a higher error in estimation. For example, the prediction of volume of distribution in rats is prone to enterohepatic recirculation, causing overestimation if allometrically scaled for the human volume of distribution [8]. There are many methods to predict the tissue-to-plasma partition coefficients (Kp) of small molecules. For example, Rodgers et al. presented a mechanistic model to predict Kp in rats, incorporating the lipid, protein binding, and ionization [9–11]. It was further extended to humans using different physiological parameters (e.g., protein and lipid concentrations and pH in plasma and tissue compartments). These predicted Kp can then be utilized to predict the volume of distribution at steady-state (VDss) using the Øie-Tozer's equation [12].

Commonly measured human PK parameters include the steady-state volume of distribution (VDss), clearance (CL), half-life (t½), fraction unbound in plasma (fu) and mean residence time (MRT). The VDss reveals the compound distribution between tissues and plasma, hence being dependent on both blood protein binding and tissue protein binding and is considered to be one of the least biased and one of the most reliable indicators of the extent of distribution [13]. CL reveals the rate at which a drug is permanently removed from the plasma [14]. The mechanisms of VDss are based on drug binding with tissue components while for CL, complex mechanisms such as metabolism and excretion via multiple pathways are involved.^15^ t½ represents the time taken for the drug concentration to reach half the initial concentration in plasma, while the MRT represents the average time spent by a drug molecule inside the *in vivo* system [15, 16].

More recently PK data has been modelled directly using 2D descriptors, ADME/PK properties as well as administered dose, as shown in Table 3. Studies have used chemical structural data to predict the volume of distribution [17, 18], the terminal half-life [19, 20], clearance [21], human plasma protein binding [22–24] and fraction unbound in plasma [25]. In particular, Schneckener et al*.* used predicted *in vitro*, physicochemical and ADME parameters and chemical structural data to classify oral bioavailability in rats with a balanced accuracy of 69.5% [26]. Obrezanova et al*.* used various machine learning algorithms, including graph convolutional networks, that rely on features derived from chemical structures, ADME and physicochemical properties to predict rat *in vivo* PK parameters of clearance (R^2^ = 0.63) and bioavailability (R^2^ = 0.55) [27]. Conformal prediction has been used for human steady-state volume of distribution predictions, using a test set of 105 compounds, achieving a twofold error of 64% [28]. Another recent study uses conformal prediction to achieve a mean prediction error between 1.4 and 4.8 for human PK parameters of fraction absorbed, oral bioavailability, half-life, unbound fraction in plasma, clearance, the volume of distribution and fraction excreted [29]. Miljković et al. established the first comprehensive protocol for the curation of human PK data and used chemical structure and administered dose for 1001 unique compounds to predict the volume of distribution in steady state and achieved an R^2^ = 0.47 [30]. These studies show that modelling *in vivo* PK parameters directly from chemical data is possible (as commonly used in predicting toxicity of molecules [31]), and this is also advantageous in the drug discovery cycle. Further, it has been shown that using predicted *in vivo* and *in vitro* data (such as Cell Painting [32]) can improve early detection of drug-induced liver injury and that biological data was predictive of drug-induced cardiotoxicity [33–35]. This is of further interest for the generative design of molecules [36] where the early estimation of *in vivo* ADME parameters is of key interest for pharmaceutical research as it can be then used early on in design, for example, in design-make-test-analyze (DMTA) cycles to prioritise compound with commonly measured PK parameters [37, 38]. While many previously developed PK models focus on parameters derived from oral administration, our study specifically builds QSAR models using intravenous (IV) PK data [39]. This choice stems from the fact that datasets based on oral dosing were originally intended for guiding healthcare professionals in understanding drug dosing, but not for establishing structure-PK relationships [39]. Oral PK data incorporate variability from absorption, first-pass metabolism, and formulation effects, making them less ideal for ML models. Many models use volume of distribution (VD) values from datasets that are frequently estimated from the terminal phase, which can add further noise to the models [39]. In contrast, IV PK data, as used in this study, reduces these sources of variability, offering a clearer understanding of the relationship between chemical structure and pharmacokinetics.

In this work, we present the first public *in vivo* PK model based on previously published datasets [40, 41]. The model used in this work, PKSmart, integrates model predicted animal PK parameters with structural and physicochemical parameters to model the human PK parameters of the steady-state volume of distribution VDss (L/kg), clearance CL (mL/min/kg), half-life t½ (h), fraction unbound in plasma fu (dimensionless) and mean residence time MRT (h). This approach is novel because it uses model-predicted, not experimentally measured, animal PK parameters as additional information to QSAR (as features) to predict human PK. This enables a fully *in silico* workflow, reducing the need for animal studies, and where the model is sufficiently predictive, captures biologically relevant cross-species patterns to enhance prediction accuracy and interpretability. The models also provide an associated fold error estimate (and a range of predictions) which is dependent on the similarity of the compound to the chemical space of the training data. PKSmart (https://broad.io/PKSmart) is freely available for integration into any design environment, with all code also downloadable for local use.

## Methods

### Data processing

#### Human intravenous pharmacokinetic parameters

Human PK data was extracted from a dataset assembled by Lombardo et al. which comprised intravenous (IV) pharmacokinetic data for 1,352 compounds.[40], [41] These parameters included steady-state volume of distribution VDss (L/kg), clearance CL (mL/min/kg), half-life t½ (h), fraction unbound in plasma fu (dimensionless) and mean residence time MRT (h). As a part of the data curation, compound SMILES were standardised which involved sanitization, normalisation, greatest fragment chooser, tautomer enumeration, and canonicalization as implemented in RDKit [42]. The standardisation process then protonates the molecule at pH 7.4 (using DimorphiteDL) by adding/removing protons to the molecule to mimic its state at the specified pH. For multiple records with identical standardised SMILES, we used median values for each endpoint (which results in mean if only two records were present). Finally, to remove molecules with molecular weight distant to the distribution of the dataset, the exact molecular weight of the compounds was calculated, and compounds with an exact molecular weight greater than 1.5 standard deviations of the mean were filtered out. For the human PK dataset, compounds with molecular weights greater than 1204.5 were removed, and for the animal PK dataset, the threshold was set at 734.3. Given that the molecular weight threshold chosen for the study was either equal to or conservative than the common range of 1000–1200 Daltons used to exclude large molecules, this also ensures that the study remains focused on small molecules. A decadic logarithm transformation was applied to all PK parameters except fu. This led to a 7.76% sparse dataset comprising of 1,283 unique compounds with 1249 VDss annotations, 1281 CL annotations, 1265 t½ annotations, 879 fu annotations and 1243 MRT annotations (henceforth referred to as the human dataset and provided as Supplementary Table S1; see Supplementary Figure S1 for distribution of data).

#### Rat, dog and monkey pharmacokinetic parameters

Distribution at steady-state (VDss), clearance (CL) and fraction unbound in plasma (fu) for intravenous (IV) dosing were compiled from another dataset assembled by Lombardo et al. which comprised 399 drugs [41]. After standardisation of SMILES using the same pre-processing as above (including protonation at pH 7.4 and a molecular weight filter of 1.5 standard deviations of the mean of this dataset) and a decadic logarithm transformation applied on PK parameters except fu, this resulted in a 34.7% sparse dataset comprising 371 unique compounds (henceforth referred to as the animal dataset and provided as Supplementary Table S2; see Supplementary Figure S2 for distribution of data).

#### External datasets

We first compiled compounds from the source of the animal PK dataset (Lombardo et al. [41]) which also contained the human VDss for 17 drugs. In addition to this, we compiled data for 51 new drugs from the literature (FDA novel drug approvals for 2021 and 2022) with VDss, CL, fu and t½ annotations [43]. For CL, we used a dataset from Yap et al. [21], who compiled total clearance in humans for 503 compounds from the literature. Out of these, we found 256 unique compounds with CL annotations that were not present in the training data used in this study (compared with standardised SMILES). Overall, we combined these datasets resulting in 315 unique compounds that were not present in the training data used in this study when compared with standardised SMILES (including protonation at pH 7.4). This dataset contained 315 unique compounds with 51 VDss annotations, 302 CL annotations, 34 fu annotations, and 38 t½ annotations, which were used as the external test set and are released as Supplementary Table S3. Additional annotations for MRT could not be identified in the literature, and hence, no external test set was available for this endpoint.

#### ATC classification and DrugBank dataset

To evaluate the coverage of our datasets in the drug space, we obtained the chemical structural information (as InChI) of 2611 approved drugs (small molecules) from DrugBank (v5.1.9) [44] as a reference point. We also obtained data on ATC classified drugs where chemical structures are available and KEGG identifiers and their classification in anatomical therapeutic chemical (ATC) (https://github.com/tonibois/KeggDrugVirtualScreening). We annotated the 2611 approved drug molecules with these anatomical therapeutic chemical ATC classification codes from the KEGG DRUG Database, resulting in a dataset of 1323 drugs with associated ATC classes.

To obtain the chemical space of the 2611 approved drug molecules, we used RDKit [43], we converted InChI to SMILES and then standardised the SMILES (including protonation at pH 7.4) and removed outliers based on molecular weight distributions below 1.5 standard deviations of the mean molecular weight in this dataset (this threshold was found to be < 918, which was also conservative to exclude large molecules). Finally, we obtained 2,304 unique molecular structures for approved drugs.

### Structural and physicochemical data

Morgan fingerprints of radius 2 and 2048 bits computed from standardised SMILES as implemented in RDKit were used as structural features. For physicochemical properties, we generated Mordred descriptors (at pH 7.4) as implemented in the Python package Mordred [45].

### Feature selection

Feature selection was performed for the human, monkey, dog, and rat datasets separately. For the Mordred descriptors, first, we used the scikit-learn [46] v1.1.1 variance threshold module to remove features having a low variance below a 0.05 threshold. Second, for all features, we calculated all pairwise correlations and removed one of each pair of features with pairwise correlations greater than 0.95. Hence, we obtained 352 Mordred descriptors for the human dataset, 379 Mordred descriptors for the monkey dataset, 383 Mordred descriptors for the dog dataset, and 386 Mordred descriptors for the rat dataset. Next, for Morgan fingerprints, we applied a variance threshold to prevent a model from fixating on less informative, minor features specific to a narrow chemical space of the dataset. By filtering out low-variance features, we ensure a model learns from more general and broadly applicable features across diverse chemical structures. To this effect, we used variance threshold to remove features having a low variance below a 0.05 threshold, resulting in fingerprints of length 152 bits for the human dataset, 207 bits for the monkey dataset, 169 bits for the dog dataset, and 153 bits for the rat dataset.

### Chemical space analysis

To assess the variability of chemical space of both the human dataset (1,283 unique compounds) and the animal dataset (371 unique compounds), we calculated the mean of 5-nearest neighbour Tanimoto similarity of each compound with the rest of the compounds in the respective datasets. Tanimoto similarity was calculated based on 2048-bit Morgan fingerprints.

To visualise the chemical space coverage of the models trained on the human dataset, we used a principal component analysis (as implemented in scikit-learn [46] v1.1.1). For this, we removed binary variables and selected 229 of the Mordred descriptors that were continuous (out of the 352 Mordred descriptors selected for the human dataset). The same 229 descriptors were used for principal component analysis to define the physicochemical space of the 1,283 unique compounds in the human dataset, the 371 unique compounds in the animal dataset and the 2,304 unique compounds in the approved drugs dataset (from DrugBank as a reference point).

### Linear regression analysis between observed animal and human PK parameters

We found 300 unique compounds common between the 1283 human dataset and 371 compounds in the animal dataset. We compared these compounds for three PK parameters VDss, CL and fu for four organisms (human, monkey, dog, and rat). We used linear regression to determine the coefficient of determination (LinearRegression() as implemented in scikit-learn [46] v1.1.1) between human and animal PK parameters.

### Model training

#### Training models on animal PK data

**Fig. 1:**
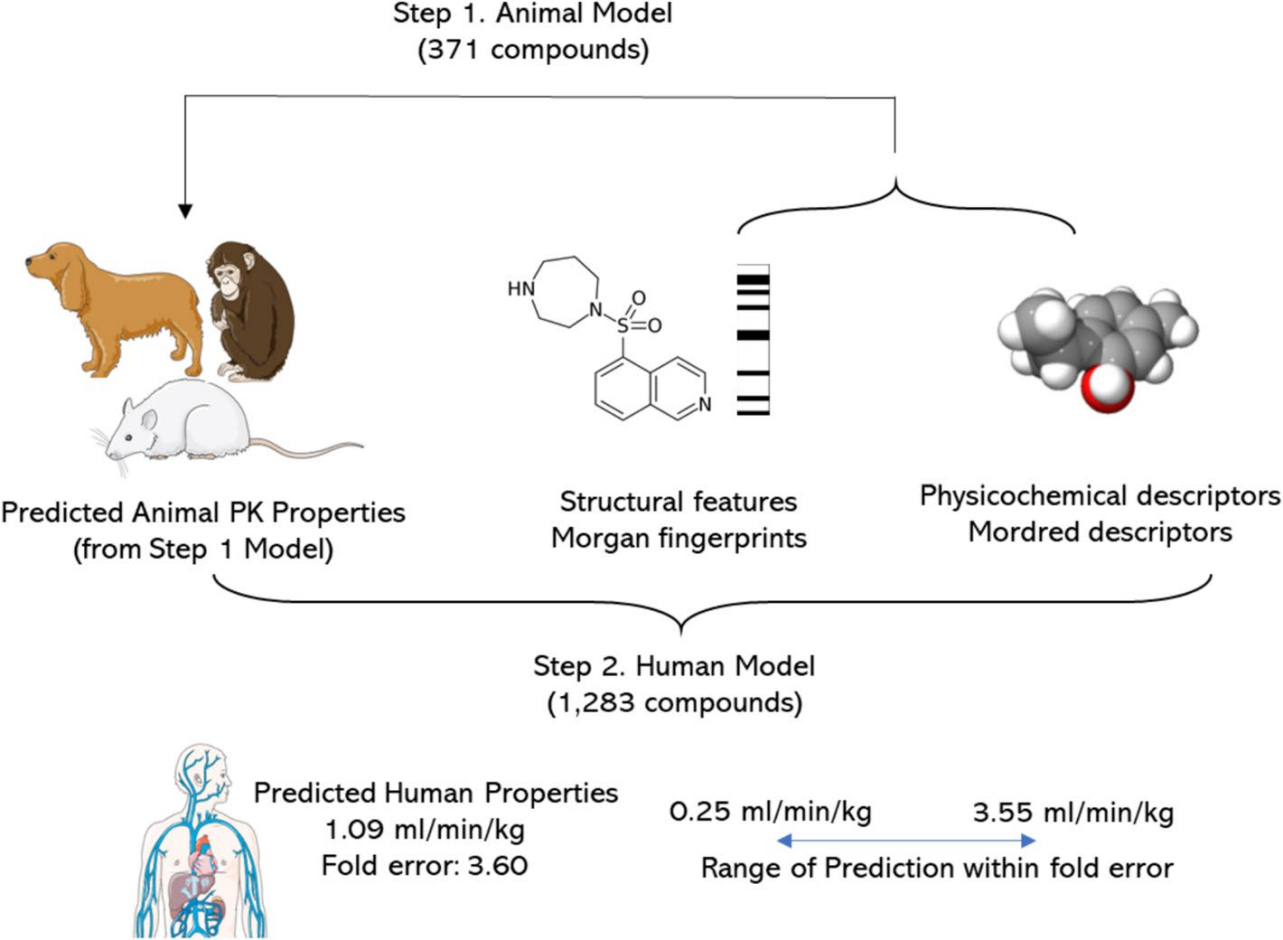
Workflow for models used in this study. First, models were trained on 371 compounds in the animal dataset to predict animal PK parameters from structural fingerprints and Mordred descriptors. Second models were trained with different combinations of the real and model predicted animal PK parameters with the structural fingerprints and Mordred descriptors for 1,283 compounds in the human dataset

We trained individual Random Forest regressor models (as implemented in scikit-learn [46] v1.1.1) for the three PK parameters from each of the monkey, dog, and rat datasets as shown in Fig. 1 Step 1. The selected Morgan Fingerprint bits and selected Mordred descriptors were standardized by removing the mean and scaling to unit variance and used as features for each of the 9 models. Each endpoint was modelled using a 5-time repeated, fivefold nested cross-validation. The data was split in the outer split into 5 folds, out of which, 4 were used to train a model and the other fold was used as the test set. For training the model we used a fourfold cross-validation. The hyperparameters were optimised during cross-validation using a grid search (Supplementary Table S4 lists the parameter grid used to optimise the Random Forest models) and the results were evaluated on the remaining test fold. This was repeated for all 5 test folds comprising the entire data. The entire process was repeated 5 times to generate different splits of data resulting in 25 test folds and corresponding 25 models. We used the lowest geometric mean fold error (GMFE) from these 25 test folds to obtain the best-performing model. The parameters of the best-performing model were used to retrain the model on the entire training data which was used as the final model.

#### Predicted animal PK data for 1,283 human PK dataset compounds

We used the 9 individual models trained to predict animal pharmacokinetic parameters of volume of distribution at steady-state (VDss), clearance (CL) and fraction unbound in plasma (fu) for all 1,283 compounds in the Human PK dataset using the models trained in Fig. 1 Step 1. These predicted animal data were used as features for modelling the human PK parameters as described below.

#### Training models on human PK data

For the five human PK parameters, the volume of distribution at steady-state (VDss), clearance (CL). half-life (t½), fraction unbound in plasma (fu) and mean residence time (MRT), we trained 9 types of Random Forest regressor models and a mean predictor (which is used as a baseline to evaluate improvement over average predictions [47]). We trained Random Forest regressor models (1) using Morgan fingerprints only, (2) and Mordred descriptors only, (3) using predicted animal data only, (4) using Morgan fingerprints and Mordred descriptors, (5) using Morgan fingerprints and predicted animal data, (6) using Mordred descriptors and predicted animal data, (7) using all three of Morgan fingerprints, Mordred descriptors and predicted animal data (Fig. 1 Step 2). In addition, we also built models (8) using a combination of predicted animal data and real animal data (where available) and (9) using Morgan fingerprints, Mordred descriptors and a combination of predicted animal data and real animal data where available. We compared these models to (10) a baseline mean predictor model that always predicts the mean of the training data. This setup allowed us to compare different combinations of features to evaluate what combination is the best to predict human PK parameters.

For each human PK parameter endpoint and model combination, we followed the same procedure used previously to build models for animal PK parameters. All features were standardized by removing the mean and scaling to unit variance before splitting the data. We used a 5-time repeated, fivefold nested cross-validation resulting in 25 test folds and corresponding 25 models from which we obtain the best-performing model with the lowest GMFE. The parameters of this best-performing model were used to train on the entire human dataset and this final model was used for predictions on the external test set.

#### Calculating fold errors

To calculate the fold error of a prediction for each endpoint, we looked at the trends for fold errors of predictions of all compounds as they appear in the 25 individual test sets. The structural similarity of each compound to their respective training data (in the particular iteration of the nested cross validation) was calculated as the mean Tanimoto similarity of 2048-bit Morgan fingerprints of the 5 nearest neighbours. For each value of structural similarity, we determined the mean fold error, and a kernel ridge regression (as implemented in scikit-learn [46] v1.1.1) was fit on structural similarity to predict the mean fold error (we removed the compounds below 1.5 standard deviations of the mean similarity to training data). The kernel used was a combination of a radial basis function kernel and a white kernel to account for the noise of the signal that was optimised by a tenfold grid search cross-validation and a scoring function to maximise R^2^ (as implemented in SciPy v1.8.0 [48]). Given the nature of structural similarity and mean fold error [49], the RBF kernel may capture non-linear relationships while by including the white kernel, we account for that noise directly in the model simultaneously, aiming to get predictions that fit the data well but are not too sensitive to the noise. Finally, using this kernel, we estimate the fold error of prediction for a query compound in the final models: we fit the numerical value of structural similarity of this compound to the entire training data for the endpoint to the ridge regressor and assigned the predicted value as the fold error of the compound for the predicted endpoint. We found that compounds with Tanimoto similarity < 0.25 were outside one standard deviation of the mean of structural similarity to the training data of the final model and this was generally where the fold error tended to be greater than 3. Thus, if a test compound had a Tanimoto similarity less than 0.25 to the training data, an alert on the compound being outside the applicability domain of the model was raised along with the prediction and the estimated fold error. Besides the performance of the best models predicting human PK parameters on each of the individual 25 test folds of the nested cross-validation, we further evaluated our model on the predictions of human PK parameters for VDss, CL, and t½ for the external test.

### Comparison to in-house AstraZeneca models

Proprietary pharmacokinetic (PK) models from AstraZeneca were procured, which included animal PK parameters for VDss, Cl, and fu for dogs and rats, and human PK parameters of VDss and fu. These proprietary models were trained features including representations of molecular structures, 2D descriptors, ADME and physicochemical properties to predict human PK data from Elsevier’s PharmaPendium PK module and internal *in vivo* animal PK data [30, 50]. PK parameters from our study were extracted using the final PKSmart models which were developed in this study independently of the AstraZeneca models. We used Pearson correlation coefficients to assess the linear relationship between predictions from the AstraZeneca models and the predictions from the PKSmart models. The strength and direction of the correlations were interpreted to determine the comparability of the PKSmart models with the AstraZeneca in-house models.

### Model evaluation

We evaluated our models based on the 2-, 3- and fivefold error, median fold error (MFE), geometric mean fold error (GMFE) and bias which were used on the decadic antilogarithm transformation on the predicted values as defined by functions in released code. With regards to direct model performance measures, we used the root mean square error (RMSE) and coefficient of determination (R^2^), as implemented in the scikit-learn v1.1.1 [46], calculated by comparing the predicted values and the log transformed true values.

For a given predicted value ${f}_{i}$ compared to the true value ${y}_{i}$, the fold error is given as:

$$FE = \left\{ { \begin{array}{*{20}c} {\frac{{f_{i} }}{{y_{i} }}, {\text{if }} f_{i} > y_{i} } \\ {} \\ {\frac{{y_{i} }}{{f_{i} }}, {\text{if }} y_{i} > f_{i} } \\ \end{array} } \right.$$

where i denotes an index running over n samples.

The 2-, 3- and fivefold error percentages were defined as the percentages of compounds for which the predicted value ${f}_{i}$ was within 2-, 3- and fivefold variabilities of the observed value ${y}_{i}$.

The median fold error (MFE) and geometric mean fold error (GMFE) can be used to provide a measure of bias while considering equally all fold errors. The average logarithmic bias (ALB) is given as,

$$ALB = \frac{{\mathop \sum \nolimits_{i = 1}^{n} log\left( {\frac{{f_{i} }}{{y_{i} }}} \right)}}{n}$$

$$GMFE = 10^{ALB}$$

Another metric we used was the bias, which gives the median error between a predicted value ${f}_{i}$ and observed value ${y}_{i}$.

$$Bias = median_{i = 1}^{n} \left( {f_{i} - y_{i} } \right)$$

We also used metrics that consider individual prediction errors; we used the RMSE which measures the distribution of prediction errors.

$$RMSE = \sqrt {\frac{{\mathop \sum \nolimits_{i = 1}^{n} \left( {y_{i} - f_{i} } \right)^{2} }}{n}}$$

The coefficient of determination, R^2^ is defined as

$$R^{2} = 1 - \frac{{\mathop \sum \nolimits_{i = 1 }^{n} \left[ {y_{i} - f_{i} } \right]^{2} }}{{\mathop \sum \nolimits_{i = 1 }^{n} \left[ {y_{i} - \overline{y}} \right]^{2} }}$$

where $\overline{y }$ is the mean of the observed data

$$\overline{y} = \frac{1}{n}\mathop \sum \limits_{i = 1}^{n} y_{i}$$

All code for these functions is released with the code on GitHub https://github.com/srijitseal/PKSmart.

## Results and discussion

In this work, we built models to predict human PK parameters of volume of distribution at steady-state (VDss), clearance (CL), half-life (t½), fraction unbound in plasma (fu) and mean residence time (MRT) using a combination of Morgan fingerprints encoding structural information, Mordred descriptors encoding physicochemical properties and predicted animal PK parameters.

### Chemical space coverage

We first aimed to explore the chemical space in the human and animal datasets to evaluate the structural variance covered, where higher variance indicates a possibility to widen the applicability domain. As shown in Figure S3, we evaluated this using the distribution of the mean 5-nearest Neighbour Tanimoto similarity (using 2048-bit Morgan fingerprints) of each compound to the remaining compounds and found that 38.9% of compounds for the human dataset (and 49.3% of compounds for the animal dataset) lie below a 0.30 threshold of Tanimoto similarity to other compounds in respective datasets. This indicates that both datasets cover a wide range of structurally varying compounds, as shown by previous studies where 0.30 was deemed a plausible likelihood estimate of a threshold for similarity searching [51, 52]. Further, the compounds used in this study for the human and animal datasets represent a wide range of physicochemical properties as shown in Figure S4 for the descriptors of molecular weight (43.0 to 1,163.6 for the human dataset, 101.0 to 709.3 for the animal dataset), clogP (−16.6 to 11.4 for the human dataset, −11.3 to 5.8 for the animal dataset) and TPSA (0 to 569.1 for the human dataset, 4.4 to 338.4 for the animal dataset). As shown in Figure S5, the datasets cover a wide range of the relevant chemical space of DrugBank. Overall, this suggests that both the human and animal datasets are representative of a broad spectrum of the physicochemical space, enhancing their use in modelling PK parameters and broadening the applicability domain of the model.

While physicochemical descriptors capture certain properties of molecules, they do not encompass the entirety of a molecule's biological activity or its interactions with biological systems. For this reason, we also compared the Anatomical Therapeutic Chemical (ATC) code distribution for these datasets. As shown in Figure S6, both the human dataset and the animal dataset covered a broad range of ATC code distribution at the top level (for 553 out of 1,283 compounds in the human dataset and for 235 out of 371 compounds in the animal dataset for which ATC annotations were available). This shows that the datasets encompass a vast array of approved drugs not only diverse in terms of chemical structures but also in their potential therapeutic applications.

### Distribution of PK parameters

We next analysed the distribution of the values for PK parameters in the human and animal datasets. Supplementary Figure S1 and S2 show the distribution of decadic logarithm-transformed data for each PK parameter and organism (human, dog, rat, and monkey) combination, except fu for which the transformation was not applied. For human CL, out of 1,281 compounds, there were 1,180 compounds with a CL ≤ 25 mL/min/kg (low CL) and 101 compounds > 25 mL/min/kg (high CL). Overall, most compounds (92.1%) in the human dataset exhibited low CL values, which is often desirable for exposure but can lead to longer half-lives that are undesirable [53]. On the whole, the datasets used in this study cover the diverse pharmacokinetic behaviour of compounds in different organisms.

### Animal PK parameters are predictive of human PK parameters

**Fig. 2:**
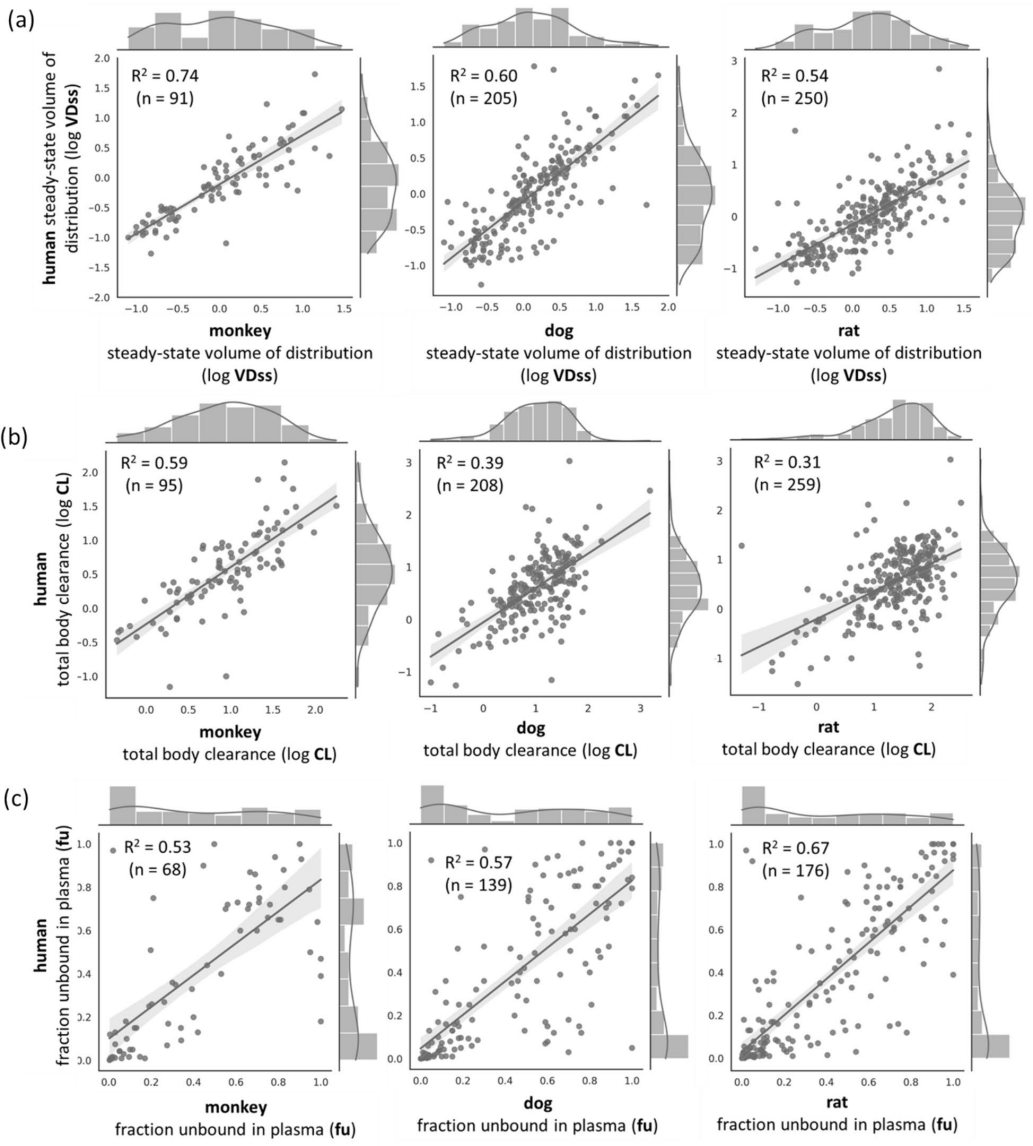
Linear regression fitting and coefficient of determination of the prediction of human PK parameters from animal PK parameters. Datasets were resampled such that the total number of unique compounds was the same for each endpoint of VDss, CL and fu

First, we analysed the animal PK data of VDss, CL, and fu for their correlation to corresponding human PK parameters to evaluate translation from animal data to human data [54]. For this, we compared the 300 unique compounds that were common in the human dataset and animal dataset. As shown in Fig. 2, we observe a linear relationship between human and monkey PK parameters (R^2^ = 0.74 with VDss for 91 compounds, R^2^ = 0.59 for CL for 95 compounds, and R^2^ = 0.53 for fu for 68 compounds) with similar trends observed for human vs dog and human vs rat PK parameters. Historically, pre-clinical compounds have been tested in animals, and only compounds that meet pharmacokinetic parameter limits in these preclinical tests proceed to clinical trials. If successful, they ultimately receive drug approval. This process creates a sampling bias in public datasets, which primarily includes drugs from clinical trials that likely have favourable animal PK assessments. Drugs that failed these preclinical tests were unlikely to advance to clinical trials, meaning the dataset lacks data on such compounds. Consequently, we mainly observe compounds that showed favourable or correlated PK profiles in both humans and animals, potentially explaining the high correlation observed in this dataset, which might not reflect experience in real-world drug discovery. While a correlation does not guarantee predictive accuracy, it does suggest similarity in some physiological mechanisms. Previously, preclinical *in vivo* PK parameters from rat have been shown to be advantageous for human PK prediction models [55]. Given this potential similarity, we incorporated predicted animal PK parameters as an additional feature space for predicting human PK parameters.

### Structural and physicochemical properties can reasonably predict animal PK parameters

We trained nine individual models for VDss, CL, and fu for each of the monkey, dog, and rat datasets using Morgan fingerprints and Mordred descriptors to assess the possibility of modelling those endpoints using the chemical structure and physicochemical descriptors alone. Table 1 shows the mean evaluation metrics of these animal PK models from the 25 test folds of the 5 times repeated fivefold nested cross-validation. Overall, the best-predicted animal PK parameters were rat VDss (mean R^2^ = 0.46, RMSE = 0.44), monkey VDss (mean R^2^ = 0.44, RMSE = 0.44), monkey CL (mean R^2^ = 0.39, RMSE = 0.42), rat fu (mean R^2^ = 0.36, RMSE = 0.26), and dog CL (mean R^2^ = 0.28, RMSE = 0.49). Since much of the drug is impacted by an organism’s physiological underpinnings, reviews have suggested a value of > 15% is likely to be a reasonable threshold for acceptable models [20]. We predicted the nine animal pharmacokinetic parameters for all 1,283 compounds in the human dataset, and these were used as features for modelling the human PK parameters as described below.

**Table 1:** Mean Evaluation metrics of animal PK models trained on Morgan fingerprints and Mordred descriptors from the 25 test folds of the 5 times repeated fivefold nested cross-validation

Organism	Endpoint	twofold error (%)	threefold error (%)	fivefold error (%)	GMFE	Bias	RMSE	R^2^
Rat	Clearance (CL)	53.89	71.18	82.26	2.50	−3.78	0.55	0.19
Rat	Volume of distribution (VDss)	56.64	75.47	89.90	2.14	0.00	0.44	0.46
Rat	Fraction unbound in plasma (fu)	58.60	70.70	79.72	2.69	0.03	0.26	0.36
Dog	Clearance (CL)	53.45	73.68	88.16	2.34	−1.06	0.49	0.28
Dog	Volume of distribution (VDss)	60.97	75.19	87.80	2.13	0.02	0.46	0.37
Dog	Fraction unbound in plasma (fu)	64.19	75.14	83.51	2.44	0.02	0.24	0.46
Monkey	Clearance (CL)	52.10	79.61	91.27	2.14	−0.53	0.42	0.39
Monkey	Volume of distribution (VDss)	60.81	75.70	87.60	2.14	0.04	0.44	0.44
Monkey	Fraction unbound in plasma (fu)	56.47	72.00	81.18	2.74	0.05	0.30	0.15

Models predicted PK parameters for rats (324 compounds), dogs (264 compounds), and monkeys (128 compounds). *GMFE* geometric mean fold error, *RMSE* root mean square error

### Model predictions of Human PK parameters in a nested cross-validation

**Fig. 3:**
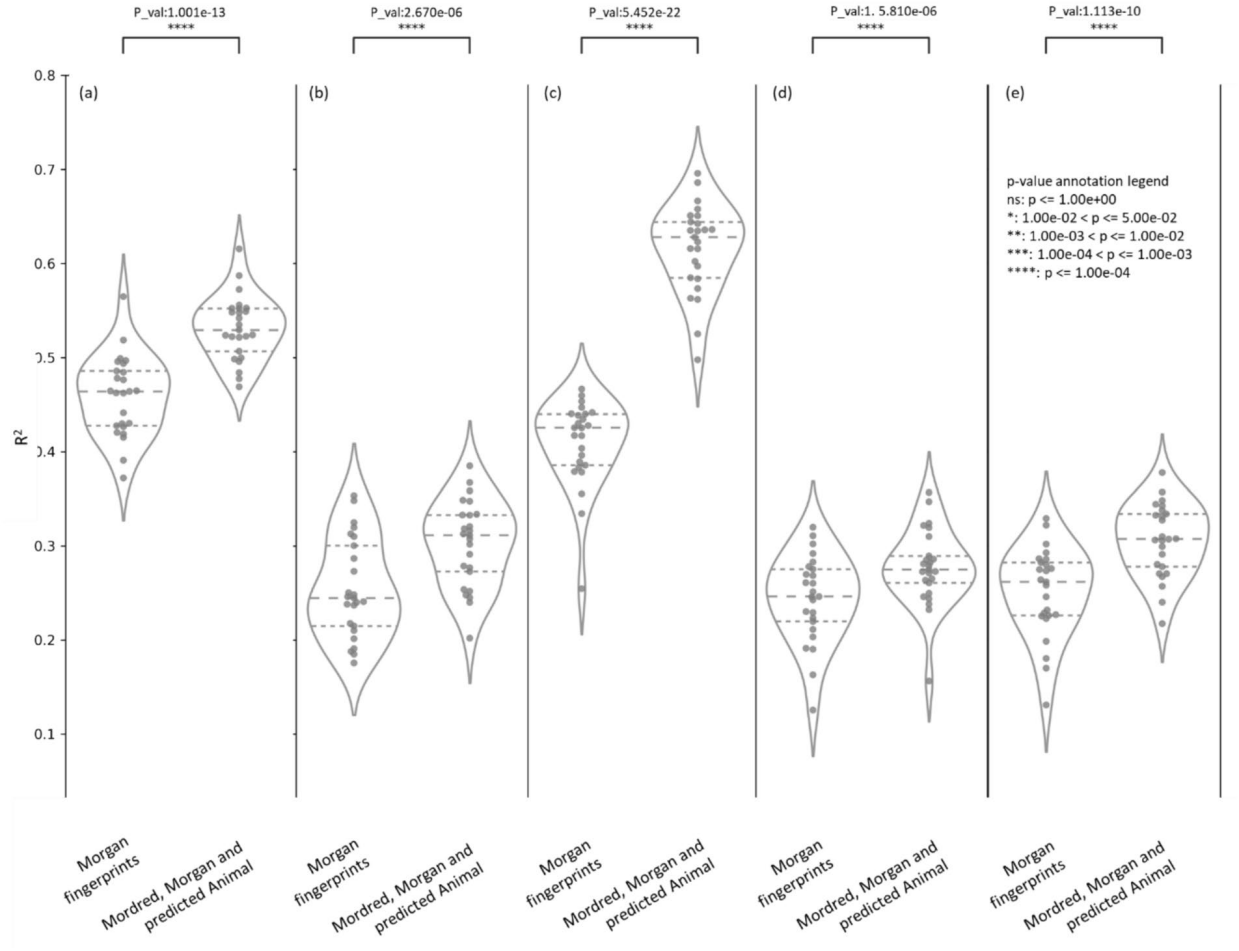
The distribution of coefficient of determination (R^2^) for models using Morgan Fingerprints versus models using a combination of Morgan Fingerprints, Mordred descriptors and predicted animal PK parameters over the 25 test folds in the nested cross-validation when predicting the five human PK parameters **a** VDss, **b** CL, **c** fu, **d** MRT and **e** t½

**Fig. 4:**
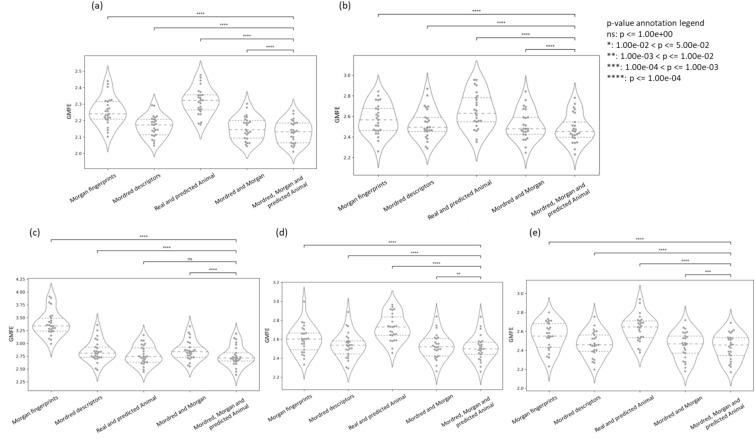
The distribution of geometric mean fold error (GMFE) over the 25 test folds in the nested cross-validation when predicting the five human PK parameters **a** VDss, **b** CL, **c** fu, **d** MRT and **e** t½

We trained 9 models to predict each of the human PK parameters VDss, CL, t½, fu, and MRT as described in the Methods Section. It can be seen from Supplementary Figure S7 that 25 test folds were comparatively dissimilar compounds (< 0.30 Tanimoto similarity) to the respective training data, as shown by the distribution of the mean 5 nearest neighbour similarity of all test compounds over the 25 test folds in the nested cross-validation. The mean predictor was found to be the worst-performing model when considering median performance metrics over all 5 endpoints (as shown in Supplementary Table S5). We found that models using all three of Morgan fingerprints, Mordred descriptors and predicted animal PK properties achieved a higher median R^2^ (R^2^ = 0.53 for VDss, R^2^ = 0.31 for CL, R^2^ = 0.63 for fu, R^2^ = 0.28 for MRT and, R^2^ = 0.31 for t½) compared to models using only Morgan fingerprints (R^2^ = 0.46 for VDss, R^2^ = 0.24 for CL, R^2^ = 0.43 for fu, R^2^ = 0.25 for MRT and, R^2^ = 0.26 for t½) as shown in Fig. 3 (further details in Table 2). These models also marginally improved median twofold errors across 25 test folds (from 55.2 to 58.0% for VDss, 48.4–50.8% for CL 47.2–55.1% for fu, 48.6–49.4% for MRT and 47.8–51.0% for t½) compared to models using only Morgan fingerprints. As shown in Supplementary Table S5, similar trends of improvement were observed when models using all three of Morgan fingerprints, Mordred descriptors, and predicted animal PK properties were compared to those using Mordred descriptors only or models using a combination of real and predicted animal PK parameters only. Further, Fig. 4 shows a significantly lower (using paired t-test) GMFE was achieved when using the models combining all three of Morgan fingerprints, Mordred descriptors, and predicted animal PK properties across the 25 test folds (median GMFE = 2.13 for VDss, GMFE = 2.45 for CL, GMFE = 2.71 for fu, GMFE = 2.50 for MRT, and GMFE = 2.46 for t½) compared to other models using structural data using Morgan fingerprints only, Mordred descriptors only, a combination of Morgan fingerprints and Mordred descriptors. Therefore, the models using all three feature spaces (Morgan fingerprints, Mordred descriptors, and predicted animal PK properties) were deemed the best-performing models to predict all five human PK parameters and are released as the final PKSmart model.

**Table 2:** Evaluation metrics for 5 human PK properties on (a) nested cross-validation and (b) the external test set using models with all three of Morgan fingerprints, Mordred descriptors and predicted animal PK properties

Endpoint	Method	twofold error (%)	threefold error (%)	fivefold error (%)	GMFE	bias	RMSE	R^2^
Volume of distribution (VDss)	Median from Repeated Nested-Cross-validation	58.00	75.20	89.20	2.13	0.03	0.43	0.53
	Held-out-test (51 compounds)	52.94	68.63	84.31	2.46	−0.02	0.56	0.39
Clearance (CL)	Median from Repeated Nested-Cross-validation	50.78	70.31	85.16	2.45	−0.26	0.53	0.31
	Held-out-test (302 compounds)	70.20	78.48	86.75	1.95	0.02	0.44	0.46
Fraction unbound in plasma (fu)	Median from Repeated Nested-Cross-validation	55.11	67.05	78.41	2.71	0.04	0.21	0.63
	Held-out-test (34 compounds)	26.47	41.18	61.76	4.12	0.06	0.22	0.26
Half-life (t1/2)	Median from Repeated Nested-Cross-validation	50.99	71.15	86.17	2.46	0.21	0.52	0.31
	Held-out-test (38 compounds)	31.58	60.53	76.32	3.31	-5.05	0.68	0.06
MRT	Median from Repeated Nested-Cross-validation	49.40	70.68	83.47	2.50	0.03	0.54	0.27
	Held-out-test (0 compounds)	N/A	N/A	N/A	N/A	N/A	N/A	N/A

*GMFE* geometric mean fold error, *RMSE* root mean square error

### Evaluation based on structural similarity and applicability domain considerations

**Fig. 5:**
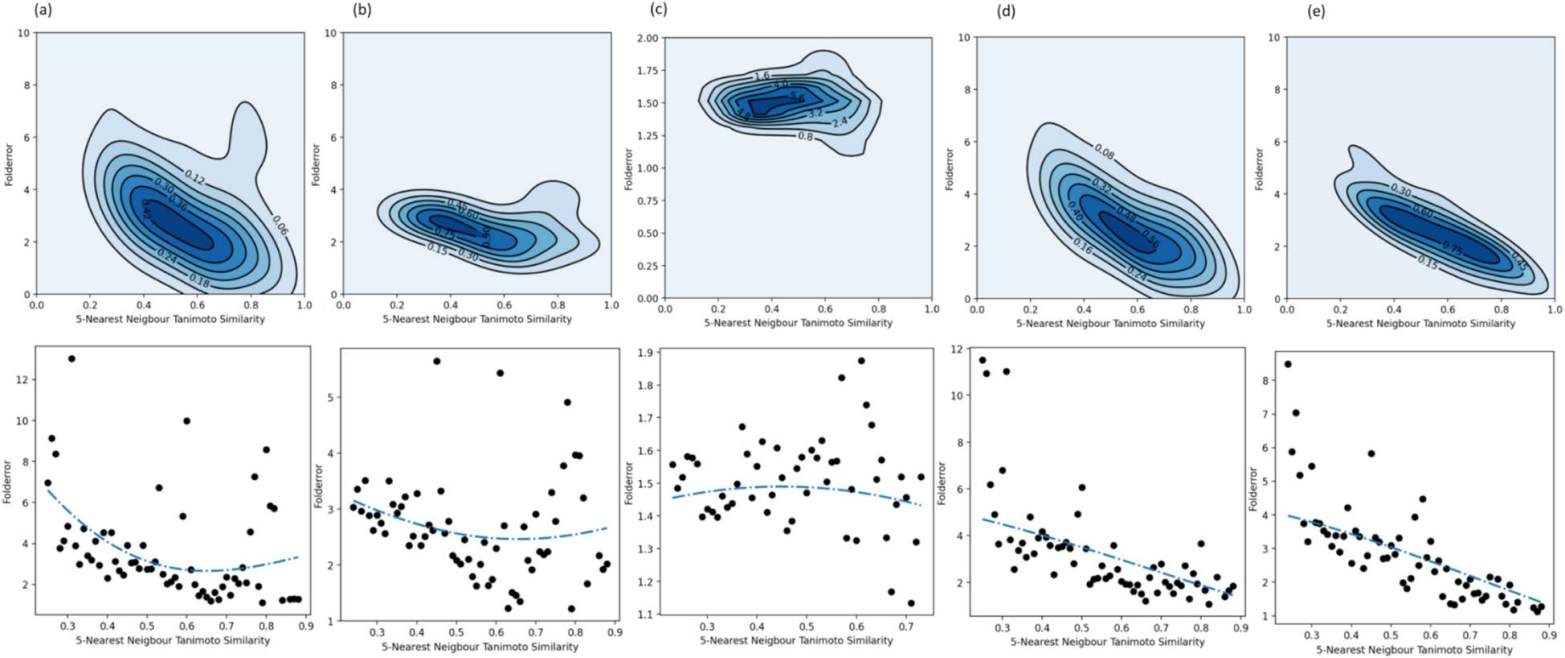
Kernel density estimate, and Kernel ridge regression show a decrease in fold error with increased structural similarity of the test compound to their respective training data during nested cross-validation for the five human PK parameters **a** VDss, **b** CL, **c** fu, **d** MRT and **e** t½

**Fig. 6:**
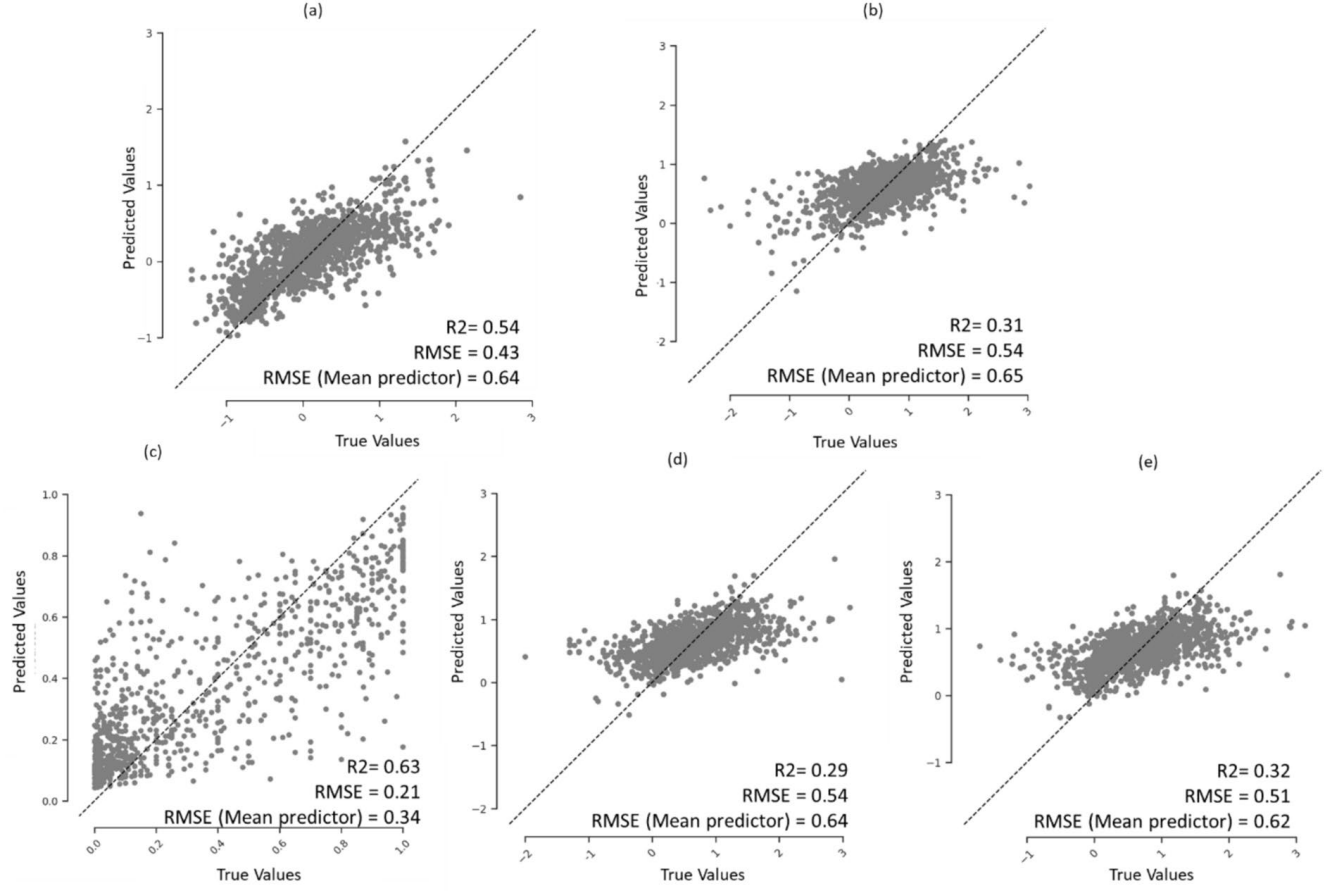
Regression plot of mean predicted PK parameters per compound over the 25 held-out test sets in the repeated nested cross-validation for the five human PK parameters **a** VDss, **b** CL, **c** fu, **d** MRT and **e** t½

We next looked at the mean fold errors for each compound that was structurally similar to the training data in the folds of the nested cross-validation for the model using a combination of Morgan fingerprints, Mordred descriptors, and predicted animal PK properties. Figure 5 shows the kernel density estimate and Kernel ridge regression curves, which, as expected show a decrease in fold error with increased structural similarity of a test compound to its respective training data. Further, we looked at the evaluation metrics using mean predictions for all compounds from all 25 folds of the repeated nested cross-validation as shown in Fig. 6. The models capture only limited variance, suggesting they detect some underlying signal in the training data but not the full complexity. This is illustrated in Supplementary Figure S8, which compares the fold error of predictions with the observed human PK parameters. The fold errors tend to be larger when the true values deviate significantly from the majority of compounds, placing them outside the prediction interval of the Random Forest models. Notably, this trend differs for fu, where model predictions are closer to or slightly higher than the true values when the true fu is < 0.2; for higher fu values, predictions tend to fall below the true values. We also note that the fu model is not trained on log-transformed data and there are fewer datapoints than other endpoints; this could be one reason why fold error is generally low when fu is < 0.2, but larger errors appear when fu exceeds this threshold.

We observed that all models consistently demonstrated a lower RMSE than the baseline mean predictor, as shown in Fig. 6 (and details in Supplementary Table S5). Evaluation metrics were improved when applying applicability domain considerations (test compounds whose mean Tanimoto similarity to the training data is more than 0.30) for all five endpoints: VDss (R^2^ = 0.63, RMSE = 0.40 for 731 compounds), CL (R^2^ = 0.43, RMSE = 0.47 for 764 compounds, fu (R^2^ = 0.66, RMSE = 0.20 for 501 compounds, MRT (R^2^ = 0.39, RMSE = 0.47 for 730 compounds) and t½ (R^2^ = 0.42, RMSE = 0.45 for 753 compounds).

Performance in PK models is often measured as the fraction within 2- or threefold error, and much less with correlation coefficients, given PK parameters such as clearance are generally about orders of magnitude and folds. PKSmart predictions are relevant to early-stage decision making, and removing, for example, high-clearance compounds from a large set of compounds and not predicting precise values in humans. Considering the predictions based on the clearance of compounds suggests that 52.5% of the 650 compounds with low clearance (< 5 ml/min/kg, i.e. < 0.70 logarithm transformed units) and 59.0% of the 978 compounds with low to intermediate clearance [56] (< 12 ml/min/kg,, i.e. < 1.08 logarithm transformed units) were predicted to be within twofold error. However, only 10.4% of the 144 compounds with high clearance (> 20 ml/min/kg, i.e. > 1.30 logarithm transformed units) are within a twofold error range. The observed discrepancy in prediction accuracy across different clearance levels suggests inherent challenges in modelling compounds with high intrinsic clearance [57].

### Model evaluation on the external test set

We next looked at the prediction compounds in the external test set that did not overlap with any of the unique compounds in the training data from the human dataset. Supplementary Figure S9 shows the pairwise Tanimoto similarity (and the contour graph) for 51 compounds for VDss, 302 compounds for CL, 34 compounds for fu, and 38 compounds for t½ in the external test dataset. The majority of pairs of compounds (over 99%) for all external datasets are structurally diverse, with Tanimoto similarity < 0.30. We compared PKSmart models with all three of Morgan fingerprints, Mordred descriptors, and predicted animal PK properties (as shown in Table 2) for evaluation metrics on the repeated nested cross-validation and on the external test set (further details of individual predictions are shown in Supplementary Table S6 for all five PK parameters). The geometric mean fold error increased in the held-out test set compared to nested cross-validation for VDss (GMFE = 2.46 in external test compared to GMFE = 2.13 nested cross-validation), fu (GMFE = 4.12 in external test compared to GMFE = 2.71 nested cross-validation), and t½ (GMFE = 3.31 in external test compared to GMFE = 2.46 nested cross-validation) but decreased for CL (GMFE = 1.95 in external test compared to GMFE = 2.45 nested cross-validation) which can be attributed to the new chemical space. Nevertheless, the models remained reasonably accurate, with an average of 45.3% of compounds within a twofold error and 62.2% within a threefold error for all four PK parameters.

### Comparison to in-house AstraZeneca models

**Fig. 7:**
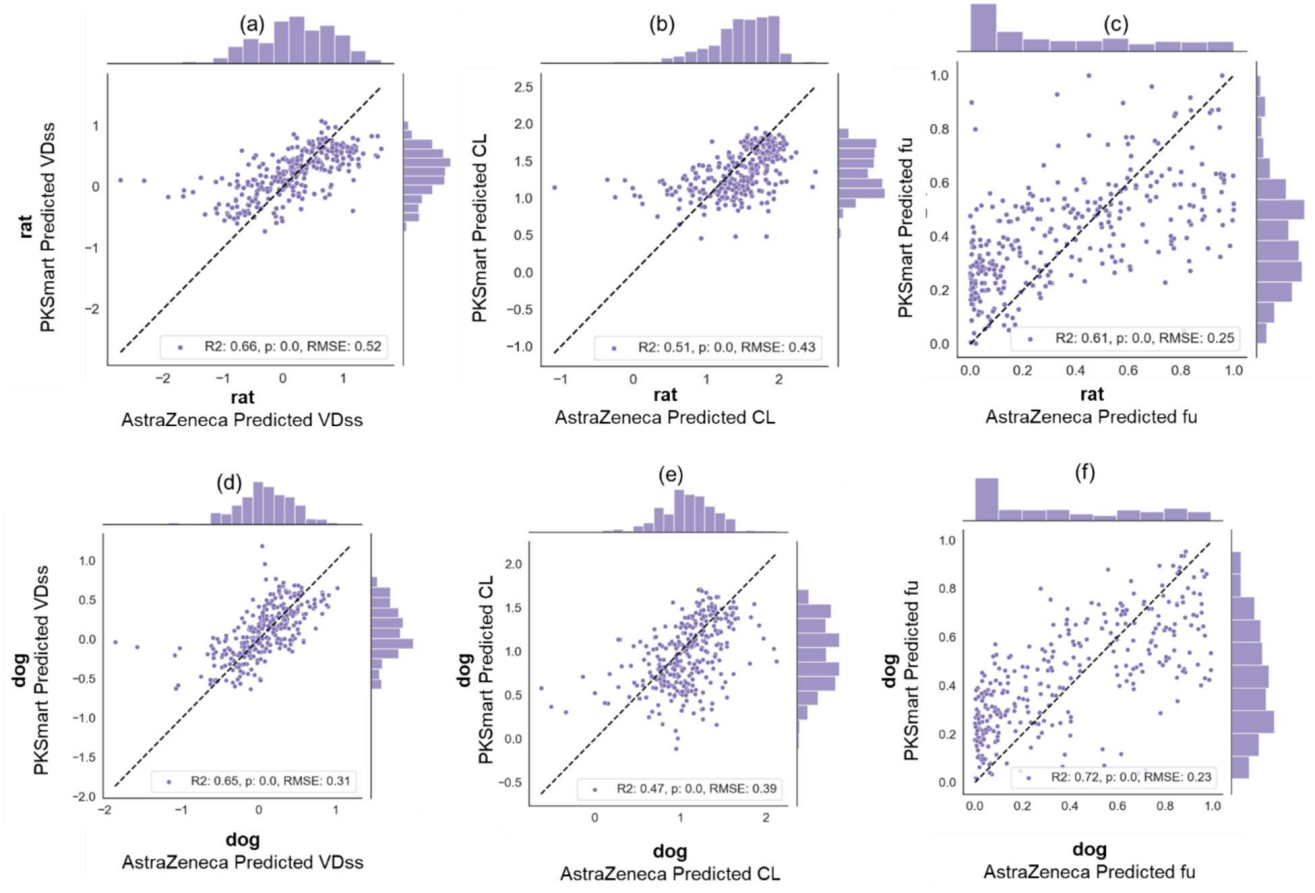
Correlation of PKSmart predictions versus AstraZeneca for animal PK parameters of rat **a** VDss, **b** CL and **c** fu, and dog **d** VDss, **e** CL and (f)fu for 315 compounds in the external test set

**Fig. 8:**
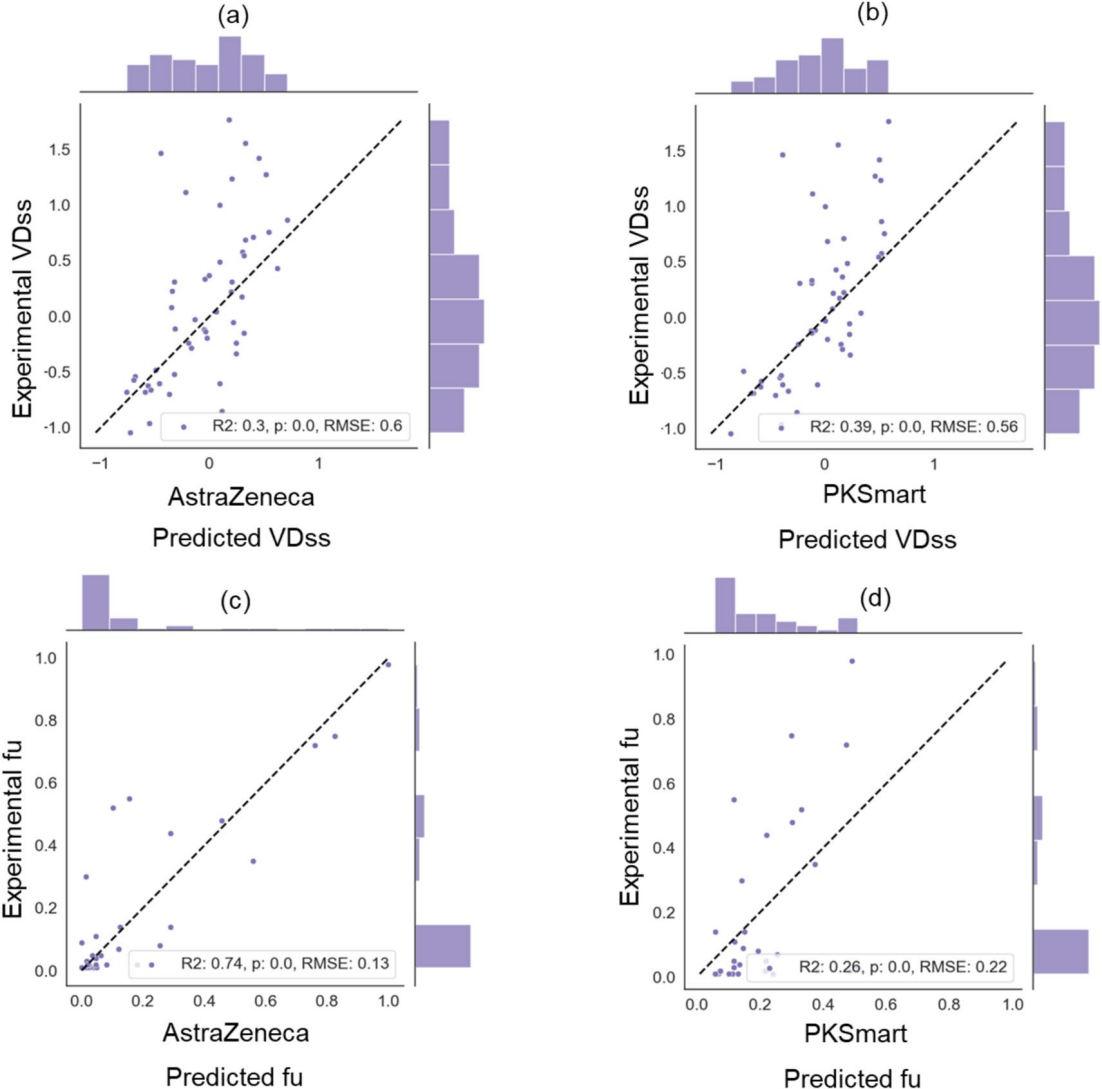
Performance of PKSmart model developed in this study and AstraZeneca model. Experimental values are plotted against the predictions for human PK parameter of **a** VDss using AstraZeneca model, **b** VDss using PKSmart model, **c** fu using AstraZeneca model, and **d** fu using PKSmart model

We compared models for human PK parameters using in-house AstraZeneca models and the predictions from PKSmart models in this study. As shown in Fig. 7, when comparing the predictions for animal PK parameters for a set of compounds (where the experimental values are not known), we see a correlation in predictions for dog and rat PK parameters for both AstraZeneca and PKSmart models (Pearson Correlation R VDss: 0.66 for rat and 0.65 for dog, CL 0.51 for rat and 0.47 for dog, and fu: 0.61 for rat and 0.72 for dog). This suggests that the PKSmart animal PK predictions correlate to the AstraZeneca's internal models for VDss, fu and Cl animal PK parameters. For both human VDss and fu, predictions from AstraZeneca models (R^2^ VDss: 0.30 and fu: 0.74) and PKSmart models (R^2^ VDss: 0.39 and fu: 0.26) are shown in Fig. 8, which suggests that while PKSmart models are well predictive of VDss; they are not as well predictive of the fu as AstraZeneca models.

### Comparison to previous literature

We next compared model performance to previously published literature. It needs to be kept in mind that models were established on very different datasets and validation methods, and hence the metrics are not directly comparable. Table 3 shows the performance of some previously published PK models [30, 58–60] Miljković et al. predicted VDss using a curated dataset of 1001 unique compounds with an R^2^ of 0.47 (RMSE 0.50) for a held-out test set, compared to this study, where PKSmart models achieved an R^2^ of 0.55 (RMSE = 0.43) when using nested cross-validation on 1,249 compounds. Further, the PKSmart models achieved an R^2^ of 0.39 (RMSE = 0.56) on an external test set of 51 compounds [30]. Iwata et al. used rat CL and chemical graph to model human CL to obtain GMFE of 2.68 (twofold error of 48.5%) in cross-validation from 788 compounds compared to PKSmart models in this study which achieved a GMFE of 1.95 (twofold error of 70.2%) when evaluated on an external test set of 302 compounds [58]. While VDss was generally easier to model, the complex biology behind CL made modelling difficult for most published models. Hence, the models developed in this study are at par with recently published literature.

**Table 3:** Evaluation metrics of previously published ML models compared to human PK models in this study trained on Morgan fingerprints, Mordred descriptors and predicted animal PK parameters

PK Parameter	Source	Features	Compounds	Validation	R^2^	RMSE	GMFE	2-fold error (%)
CL (human)	Iwata et al. [58]	Chemical Graph + rat CL	748	Cross Validation	–	–	2.68	48.5
CL (rat)	Kosugi et al. [60]	Structural features and physicochemical descriptors	1114	Cross Validation	0.56			71.9
CL (human)	Wang et al. [59]	Molecular descriptors	1268	Cross Validation	0.88	0.10		
CL (human)	Current work	Morgan Fingerprints, Mordred Descriptors and Animal PK	1281	Median from Nested Cross Validation	0.31	0.53	2.45	50.8
CL (human)	Current work	Morgan Fingerprints, Mordred Descriptors and Animal PK	302	External Test set	0.46	0.44	1.95	70.2
VDss (human)	Fagerholm et al. [28]	Signatures molecular descriptor	105	External Test set	0.65			64
VDss (human)	Miljković et al. [30]	2D, ADME/rat PK, Dose	1001	Held out test	0.47	0.5		48.5
VDss (human)	Current work	Morgan Fingerprints, Mordred Descriptors and Animal PK	1249	Median from Nested Cross Validation	0.53	0.43	2.13	58.0
VDss (human)	Current work	Morgan Fingerprints, Mordred Descriptors and Animal PK	302	External Test set	0.39	0.56	2.46	52.9

*GMFE* geometric mean fold error, *RMSE* root mean square error

### Limitations of this study

We note that PKSmart does not need to be individually parameterized for each compound and with comparable fold errors to the methods described. For example, it would be logical to assume that an approach that is tailored to a specific compound using experimentally derived data to predict VDss would outperform a model that is only using chemical structure, however, the ability to predict for an compound without needing to be an expert in PBPK modelling offers some key advantages in the early stages of drug discovery where experimental data may not be readily available [61] (especially if the compound has not been synthesized yet) and we want to compare across a few hundred molecules in a few chemical series. With PKSmart, we can predict PK instantaneously based solely on chemical structure, which makes our ML-based approach fast and easy to apply, even at the point of design.

PKSmart learns from the training data, which contains mostly drugs exhibiting linear pharmacokinetics, which is then appropriate for an approximate estimation of PK parameters (for example, clearance) for broad prioritization in an early stage in most therapeutic scenarios. However, we acknowledge PKSmart may be limited for drugs exhibiting non-linear kinetics [62]. Where similar compounds behave with non-linear kinetics, this behaviour could be picked up, but as a predominantly QSAR model, PKSmart does not account directly for kinetics. The intention for PKSmart is to be used in the early stages of drug discovery to provide rapid estimates of PK parameters. For compounds suspected of non-linear behaviour, we recommend users interpret predictions with appropriate caution and consider experimental validation.

This work is concerned with intravenous PK parameters only, which aims to use QSAR to predict the relationship between chemical structures and pharmacokinetics into account without taking oral absorption into account [39–41]. This approach was suitable here for the prospective application of PKSmart in early drug discovery.

While PKSmart demonstrates strong performance across multiple human PK parameters, its practical deployment requires careful consideration of its applicability domain and predictive confidence. The use of fold-error thresholds and uncertainty flagging is a useful step toward responsible application; however, limitations remain, particularly in extrapolating to novel chemical space [63] with sparse human PK coverage. A core limitation, as in many ADME/PK modeling efforts, is the scarcity of high-quality human PK data for training [64]. In contrast, industrial databases often contain large and chemically diverse sets of animal PK data (such as Elsevier’s Pharmapendium). Integrating such proprietary datasets into the PKSmart could significantly expand its applicability domain and improve model generalizability, especially for structurally novel compounds.

Future extensions of this approach could involve disentangling the individual contributions of predicted animal PK parameters to each human endpoint (e.g., using SHAP or ablation studies), or selectively weighting them based on species relevance. Furthermore, future model development could incorporate representation-learning, meta-learning or transfer learning strategies [65], enabling the models to adapt to new compounds or endpoints with limited human data. Another promising avenue is to explore uncertainty-aware active learning, where PKSmart could identify compounds that would most benefit from additional *in vivo* validation, thus closing the experimental-computational loop [66].

### Publicly available tool PKSmart

All code used to present results in this work is released publicly at https://github.com/srijitseal/PKSmart. All generated data from this code is further released at Zenodo (10.5281/zenodo.10611606). The final model that combined all three of Morgan fingerprints, Mordred descriptors and predicted animal PK properties was released as a python/streamlit-based web-hosted application PKSmart at https://broad.io/PKSmart (also accessible via https://pk-predictor.serve.scilifelab.se/). Users can access the application using a web browser or locally with all code available via Zenodo at 10.5281/zenodo.10611606.

## Conclusion

In this proof-of-concept, we used structural fingerprints, physicochemical descriptors, and predicted animal PK parameters to develop models for human PK parameters. We have developed a publicly available tool using machine learning to predict these PK parameters and this is the first work that publicly releases PK models on par with industry-standard models. The web-hosted application developed in this study allows the user the predict the PK parameters from the input of chemical structure only and returns a range for each prediction with an estimated fold error of a compound based on the similarity to training data. This helps impart some understanding of the applicability domain of the models. Integrating animal PK features from across a range of species could therefore be used for fit-for-purpose and improved PK prediction in drug discovery. Such models can then be integrated into DMTA cycles to facilitate compounds with desirable PK parameters in the early stages of drug discovery. Future studies could further explore the impact of individual animal PK parameters in predicting human PK parameters, where the greater availability of public data could significantly improve predictive models. The web-hosted application PKSmart can be accessed at https://broad.io/PKSmart via web browser and with all code downloadable for local use at 10.5281/zenodo.10611606.

## Supplementary Information

Supplementary material 1.

Supplementary material 2.

## Data Availability

We released the Python code for our models which are publicly available at https://github.com/srijitseal/PKSmart and code ready for local implementation is available via Zeno-do at 10.5281/zenodo.10611606. PKSmart is freely available at https://broad.io/PKSmart (also accessible viahttps://pk-predictor.serve.scilifelab.se)
